# Male Infertility, Precision Medicine and Systems Proteomics

**Published:** 2018

**Authors:** Niloofar Agharezaee, Mehrdad Hashemi, Minoo Shahani, Kambiz Gilany

**Affiliations:** 1- Department of Genetics, Faculty of Advanced Science and Technology, Tehran Medical Sciences, Islamic Azad University, Tehran, Iran; 2- Faculty of Advanced Science and Technology, Tehran Medical Sciences, Islamic Azad University, Tehran, Iran; 3- Reproductive Biotechnology Research Center, Avicenna Research Institute, ACECR, Tehran, Iran; 4- Metabolomics and Genomics Research Center, Endocrinology and Metabolomics Molecular Cellular Sciences Institute, Tehran University of Medical Sciences, Tehran, Iran

**Keywords:** Infertility, Male, Precision medicine, Proteomics

## Abstract

Precision medicine (PM) is an approach that has the power to create the best effect and safety of medicine and treatment with the least side effects for each person. PM is very helpful as sometimes due to inaccurate or late diagnosis or toxicities of the drugs irreversible side effect for patient’s health are generated. This seemingly new and emerging science is also effective in preventing disease, due to differences in the genes, environment, and lifestyles of any particular person. PM can be a prominent criterion in infertility research. To achieve this goal, there should be information from a healthy human body, including genetic and molecular information. A PM is an evolution in health care, which is very helpful even economically. The guarantor of the PM success is the examination of the molecular profile of the patient, including genes, proteins, metabolites, *etc*. Therefore, genomics, proteomics, and metabolomics-based techniques are very important in this regard. Unfortunately, despite extensive studies on PM practice in various fields, male infertility has remained unresponsive. Given that around 20% of couples around the world suffer from infertility, and almost half of them are related to men’s problems, the PM approach has a high potential for male infertility. In this study, with the help of proteomics and metabolomics, PM information on male infertility was explored.

## Introduction

Precision medicine is a new technology for managing patient health, which is used to achieve the best results in the management of the disease. This initiative makes prevention, diagnosis, and treatment more accurate than ever with minimal side effects and thus saves time and money in health care. Physicians can have a custom treatment for each patient (1, 2). PM in the last decade could cause significant advances in the clinical management of many diseases, including cancer, cardiovascular disease, diabetes, autoimmune diseases, inflammatory diseases, orthopedics, neurodegenerative diseases, and infertility (3–6). Currently, most medications have been approved according to their performance in a population of patients, meaning that the average positive response in a group to a new drug product qualifies for clinical trials. However, in the future, medications will provide personalized solutions based on the specific needs of each patient. The reason is that each person responds differently to the drug. The reason is that the drug response in a patient may be negative, or in another person has a significant response, or even leads to dangerous complications in another patient. Therefore, the concept of “one medicine for all patients with the same disease” in the new era is an inadequate expression. Thus, the science should go toward precision medicine (PM), which aims to give the right medicine at the right time to a specified patient. The design of the drug should be based on genotype, living environment and other individual characteristics of the patient that may affect the therapeutic response. Omics branches have entered PM field *e.g*. genomics, proteomics and metabolomics-based technologies have facilitated the drug development. Omics technologies can advise physicians to anticipate the effectiveness or likelihood of drug complications prior to prescribing. Therefore, PM is the perfect spot for integrating new medical biotechnology to manage diseases and patients. PM forces pharmaceutical companies to produce more effective drugs with the least side effects. Physicians with access to genetic information can optimally and safely use existing drugs. Patients with knowledge of their genetic characteristics, can manage their health in a variety of settings (7).

In the field of male infertility, there is still room for improvement of drug development. In this study, PM and its potential targeted treatment of male infertility were introduced to achieve optimal diagnostic and therapeutic advances for each individual. By combining the molecular and clinical picture with the help of omics technology, a more precise understanding of male infertility will be described (8). A proposed PM is based on systems proteomics on male infertility. A review of antioxidant therapy and discovery of male infertility biomarkers is provided in this paper.

### Precision medicine:

With the launch of the human genome project in the late 1980s, the researchers hoped that a change would occur in healthcare, given the genetic differences of each person with regard to their molecular profiles (9). Therefore, medical science began to change with the goal of preventing the disease from reactive medicine to proactive predictive medicine (10). A new era in the prevention, diagnosis, and treatment of individuals has started. In this course, patients are not classified according to the type of disease, but the goal is to have each patient a personalized treatment (10). The terms personalized medicine, precision medicine, stratified medicine and P4 medicine (system medicine) are used interchangeably (11, 12). That is, medical treatment for each patient is based on his personal characteristics (13), because each person is different based on genetic information, epigenetics, environment, lifestyle, and medical history (14, 15). However, it does not mean that medicines or medical devices are unique to a patient. On the other hand, they are aimed at categorizing individuals into groups that differ in their sensitivity to a particular disease and have a prognosis that may be the cause of the response to a special treatment (13). With optimal prevention and treatment, fewer costs and side effects are created (16). However, since the term “Personalized medicine” is sometimes misinterpreted in the sense that a person has to be treated in a unique way, the term “precision medicine” is preferred, which was raised in the IOM (The Institute of Medicine) committee (9, 13). The notion that we are probably able to supply the right drug to the proper individual at the right time, has encouraged researchers’ in-depth efforts. Through these efforts, a new dimension by the precision medicinal drug initiative, was announced by President Obama in 2015 (17). In recent years, many countries have implemented different precision medicine initiatives (PMIs) nationally including USA, China, Australia, Qatar, South Korea and some countries in Europe (18). This indicates those countries have recognized the importance of this approach at the national level, both in terms of public health and economics. This science can progress well if recurrent clinical conditions are redefined for specific molecular and genetic factors with potential benefits for treatment and prevention. Therefore, physicians can recognize problems more accurately by molecular diagnosis and different responses that are obtained. The estimate of today’s drug treatment according to the published report by the U.S. shows that only a few drugs have effects on 60% in the best case (9, 19).

Therefore, PM introduction will introduce an improvement in above rates. The inadequacy of current medications to save patients’ lives can be a major reason for turning to the medical care.

**Figure 1.**
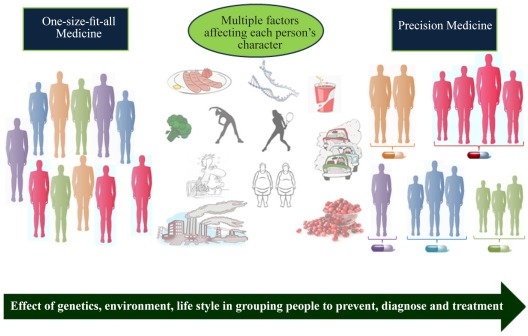
Comparison of differences in the biological characteristics of each individual and the differences in his classification

Of course this idea, which seems to be new, goes back to many years ago. PM concepts were expressed by the greatest Persian physician Avicenna (AD 980 to 1037) about 1000 years ago. “Every drug will have different effects on the bodies of individuals and individual organs,” he said, confirming the effect of a drug in two different times. In the body of an individual, as well as in an organ at different times, the effect can be various (20). The idea of PM is that individual fluctuation is considered when choosing a patient’s treatment strategy (21). The objective of exact solution is to locate the correct pharmaceutical and the correct measurement for every patient without any rate of failure. One of the drawbacks of traditional therapies is that the patient does not get effective treatment on time. It is imperative that the healthcare community take the move away from a one-size-fits-all approach to taking care of patients for rapid and effective treatment with maximum benefit and the least harm (Figure 1) (22).

One of the challenges that PM faces, apart from ethical issues, is the uniqueness of studies and, consequently, their less relevance to clinical component. Omics technology can help to better apply PM. For example, the fields of genomics and bioinformatics born in conjunction with Human Genome Project (HGP) have been instrumental in PM examination. However, the studies on the post-translational modifications (PTMs), which shows drugs effects are handled by a few experts (23). This shows a lack of expertise in the integration of omics technology for the benefits of PM. Developments of multidiscipline studies are needed for clinical propose, because of the unique biological characteristics of each person such as genomics, transcriptomics, proteomics, metabolomics, epigenomics, pharmacogenomics (1, 24–26). As an example, recent research has shown that transcript levels by themselves are not sufficient to predict protein levels in lots of situations. In order to understand the biological processes and the genotype-phenotypic relationships, it is important to integrate all necessary omics levels (27).

### System proteomics and PM:

While the roots of PM are genomic medicine, they go beyond the genetics and fully cover the physiology of the cell and its complexity (28). Proteins are the important building blocks of the cell, preserving a structural, functional and regulatory role, being accordingly accountable for the orchestration of biological functions of the cell. It is a well-known fact that all available drug targets are proteins (29). The term proteomics, which means a high-throughput analysis of proteins, was introduced in 1997 (12, 30, 31). The results of proteomics include the protein content or a variety of proteins expressed in different conditions. Therefore, the interpretation of these results in any person can be an effective step in the diagnosis and treatment of that person in PM. Proteomics is a developing field because of the complexity of proteome (32, 33). However, despite the fact that the field of proteomics offers a great deal of promise in these areas, few tests or valid markers have come to the clinical laboratory (34). Proteomics takes a real-time snapshot of a patient’s protein profile for the duration of the disorder (29). The data set from the various tissues of healthy samples is the basis of clinical proteomics. By comparing patient samples with this database, scientists associate proteome changes with diseases. Using these findings in clinics helps physicians analyze patient proteins by comparing them with healthy archives. Proteomics has the potential for an early detection and rapid response from patients. Additionally, proteomics is cost effective. With this potential approach, we will increase the accuracy of the diagnosis and save time and cost ahead (35). Certainly, a good diagnosis is a great value to start a treatment.

**Figure 2.**
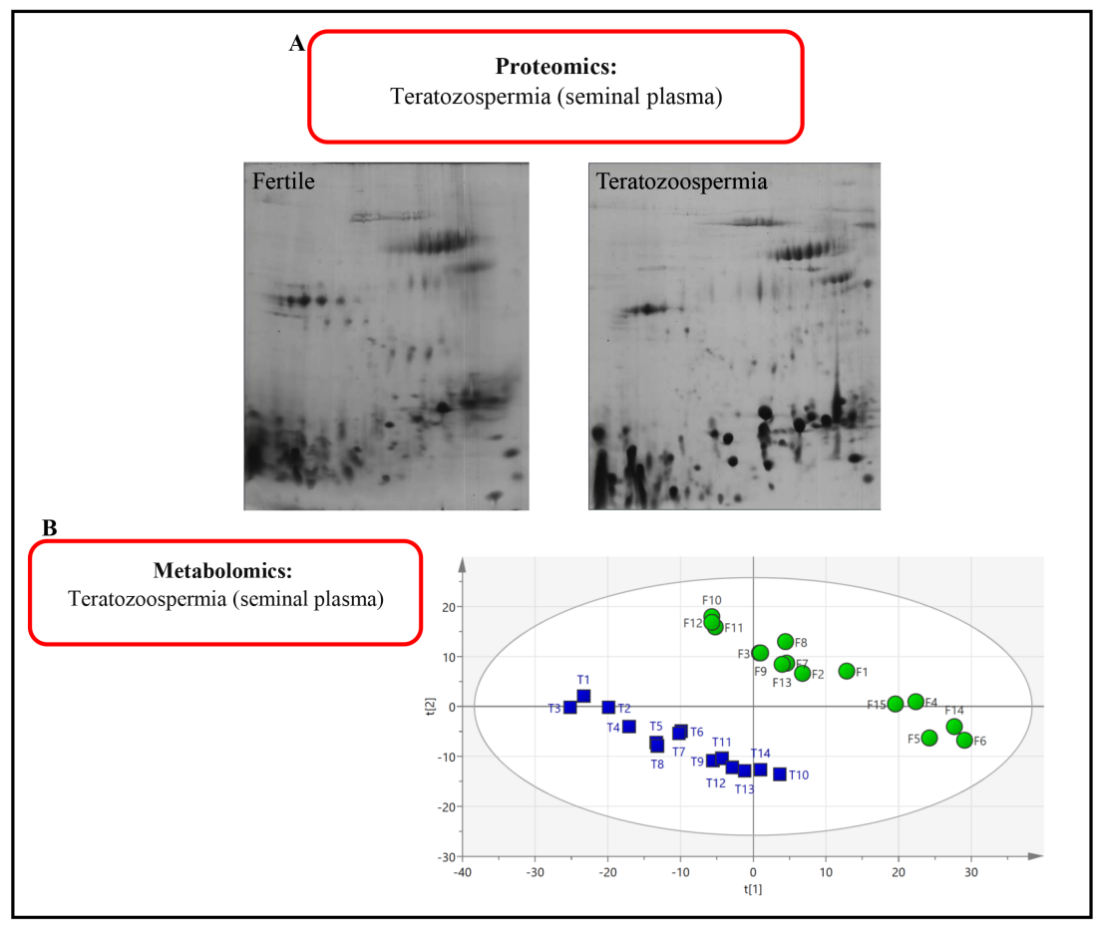
A: Comparison of the proteomics of seminal plasma in a fertile person and patient with teratozoospermia. B: The same metabolomics comparison. F: Fertile; T: Teratozoospermia

Characterization of the human plasma proteome has emerged as a major purpose inside the proteomics area. However, it is a challenging proteome of all human body (36). As an example, in the case of male infertility, there has not been much work on the disease of teratozoospermia (Abnormal sperm morphology). Our primary proteomics human seminal plasma (HSP) analysis of those patients compared to fertile men is shown in figure 2. Two-dimensional electrophoresis (2-DE) was applied to analyze proteome teratozoospermia HSP. HSP is a valuable biological source. However, it is not used in the diagnosis of infertile men, despite the fact that the sample preparation of seminal plasma is easy (37). As shown in figure 2a, novel biomarkers were identified in 2-DE of HSP from teratozoospermia compared to fertile men.

Metabolomics is a relatively new “omics” high-throughput field. It has wide application. However, it has limited scope in male infertility (32). This technology was successfully applied to analyze HSP of infertile men (32, 38–41). Most recently, metabolomics was used to analyze HSP of teratozoospermia. Our primary results using nuclear magnetic resonance spectroscopy (NMR) to study HSP of teratozoospermia metabolome shows a clearly separate profile from fertile men (Figure 2b).

### PM and male infertility (Antioxidant therapy):

About 20% of couples are estimated to be infertile (38). Infertility is a disease that can have a negative effect on the mental health of couples. Male factor infertility affects about half of the infertile couples (32). There is a high incidence of various diseases in infertile men including cardiovascular disease and cancer (42–45). Genetic causes can have direct effects on infertile men. Therefore, genetic research is nowadays a very effective way of determining the prognosis and treatment of infertile men (46). For men, diagnostic tools are limited to a standard analysis of semen. This basic test provides a physician with an overall estimate of infertility (47). In the diagnosis of infertile men, the typical parameters of the semen analysis are sperm motility, sperm morphology, concentration or count. To the best of our knowledge, there is no parameter for analysis of the seminal plasma (37), despite the fact that the seminal plasma contains rich molecules made by male reproductive glands (37). HSP is very suitable for reflecting the anti-oxidant therapy studies. Oxidative stress (OS) plays an important role in sperm performance (48, 49). OS is a result of the imbalance between reactive oxygen species (ROS) and antioxidants. This imbalance can have harm on spermatozoon which can lead to male infertility (50–54). It is estimated that 25%–40% of infertile men have significantly higher degrees of ROS in their semen compared with fertile men (48). Therefore, antioxidants are considered in the treatment of male infertility in the quest of accurate PM goals. Antioxidant therapy program according to the PM will help the reproductive health of couples as well as improve their understanding of their infertility (55). However, the collection of strong and high-quality data on molecular level of antioxidant therapy is not available. It is shown in a few studies that antioxidant therapy has an improving effect on the resolution of spermatic dysfunction in the infertile men (48, 51, 56–59).

In order to understand the antioxidant therapy effects in the asthenozoospermic men (Sperm with slow motility), a pharmacogenetic study was performed. The semen samples were evaluated for each sample based on WHO standard and criteria. Our antioxidant in the study was vitamin E, selenium and folic acid (Vit E 400 *IU/day*+ Selenium 60 *mcg/day*+ Folic acid 5 *mg/day*). A study was conducted on 10 affected men. The men of asthenozoospermia with the following conditions were included in the study; 1) The age range between 20 to 45 years, 2) Having no chronic disease, 3) Absence of supplementation of vitamin E and folic acid in the last 3 months, 4) Not taking another drug for 3 months.

Our primarily results of sperm parameters after three months of treatment are presented in table 1. As shown in the table, antioxidant therapy does not show a significant improvement in sperm parameters. Therefore, a zoom in view is required at the molecular level such as transcriptome, proteome or metabolome. This primary result shows that how significant is the PM concepts application in the treatment of male infertility.

**Table 1.** The results of three-month antioxidant therapy in patients with asthenozoospermia. Comparing the results before and after taking the drug

**Patient no.**	**Count**		Motility^*^		**Normal Morphology (%)**	

	**Before**	**After**	**Before**	**After**	**Before**	**After**
**1**	20×10^6^	11×10^6^	a=0; b=20	a=0; b=35	5	3
**2**	40×10^6^	40×10^6^	a=0; b=10	a=0; b=10	5	5
**3**	20×10^6^	16×10^6^	a=0; b=20	a=3; b=20	5	4
**4**	16×10^6^	17×10^6^	a=0; b=20	a=0; b=20	6	6
**5**	20×10^6^	26×10^6^	a=0; b=30	a=5; b=35	4	5
**6**	48×10^6^	48×10^6^	a=0; b=30	a=0; b=30	4	4
**7**	28×10^6^	35×10^6^	a=5; b=25	a=0; b=35	5	6
**8**	34×10^6^	38×10^6^	a=0; b=30	a=0; b=25	4	4
**9**	25×10^6^	16×10^6^	a=0; b=30	a=0; b=30	4	3
**10**	32×10^6^	38×10^6^	a=0; b=25	a=0; b=35	5	4
**Average**	28.3×10^6^ ±10×10^6^	28.5×10^6^ ±12.9×10^6^	a=0.5±1.58; b=24±6.58	a=0.8±1.75; b=27.5±8.58	4.7±0.67	4.4±1.07

* a: Motility a (%; fast progressive), b: Motility b (%; slow progressive)

There is still a need for a lot of research on male infertility and PM. HSP as a novel source in the research of male infertility and PM can be used in this regard.

## Conclusion

Many causes of male infertility are still unknown. Therefore, genomics, transcriptomics, proteomics, and metabolomics are very effective ways to investigate the molecular level of the disease. Physicians can use PM potential for the better effectiveness of treatment with early diagnosis and effective medications. Additionally, each person’s unique treatment conditions should be taken into consideration. The link between medication and male infertility, including antioxidant therapy, can be highly effective. Furthermore, it needs a further research in this field and the researchers and physicians must collaborate more. This can dramatically help develop new therapies for male infertility. Although this is a long way, the results are promising.
